# Airflow dynamics in obese minipigs with obstructive sleep apnea

**DOI:** 10.1016/j.heliyon.2020.e05700

**Published:** 2021-01-19

**Authors:** Zi-Jun Liu, Tiffany Do, Hanson Fong

**Affiliations:** a Depts. Orthodontics & Oral Health Sciences, School of Dentistry; b Dept. Material Sciences and Engineering, College of Engineering, University of Washington, Seattle, WA, 98195, USA

**Keywords:** Obstructive sleep apnea, Computational airflow dynamics, Pharyngeal airway, Respiration, Obesity, Pigs, Health sciences, Respiratory system, Physiology, Anatomy, Eye-ear-nose-throat

## Abstract

**Objectives:**

Obstructive sleep apnea (OSA) is associated with anatomical restrictions of pharyngeal airway, but the mechanism of airflow dynamics in OSA is largely unknown. This study utilized computational flow dynamics (CFD) to build a 3D model of the pharynx and to test the hypothesis that an increased restriction in the pharynx in OSA/obese minipigs leads to higher resistance, which in turn creates turbulence to induce temporary blockage of pharyngeal airway patency.

**Design:**

Of five 9-11-months-old Yucatan minipigs, 3 were non-obese (BMI<35) and two obese (BMI>51). After natural sleep monitoring using BioRadio system, pigs were sedated to collect MRI images and airflow parameters. The MRI images were processed to create 3D configurations of pharynx. These 3D configurations were meshed to create finite element models (FEM) of CFD. The obtained airflow parameters were input into the configurations to identify turbulent airflow and its location.

**Results:**

Heavy snoring and multiple >5s hypopnea/apnea episodes (AHI = 32–35) were identified in both obese minipigs during sleep. Compared to the non-obese/non-OSA controls, obese/OSA minipigs showed much lower respiratory tidal volumes and inspiratory airflow speed. FEM simulation found that turbulence was not present in the pharynx in either model. However, a 25% increase of airflow velocity was observed at the narrowest part of the nasal pharynx in the obese/OSA minipig model.

**Conclusions:**

Despite the narrower pharyngeal airway and the higher velocity of airflow, FEM simulation indicated that turbulence was not produced in the obese/OSA minipigs.

## 1 Introduction

Obstructive sleep apnea (OSA) is characterized by the repeated narrowing of the pharyngeal airway during sleep. The blockage of oxygen flow due to the collapse of the pharyngeal airway requires patients to wake up in order to regain airway patency. In addition to decreasing the quality of sleep, OSA has been closely associated with many dangerous consequences including an increased risk of diabetes, heart rate, blood pressure, liver problems, and complications with medications (Marin et al., 2005). One of the biggest risk factors of OSA is obesity, as it contributes to expanding the mass of structures around the pharyngeal airway and in turn, narrowing the airway passage. Both animal and clinical studies have revealed that obesity not only increase the sizes, but also accumulates adipose tissue in the tongue and soft palate, which positively correlates with the severity of OSA (Brennick et al., 2014; Kim et al., 2014; Schwartz et al., 2008).

**Figure 1:**
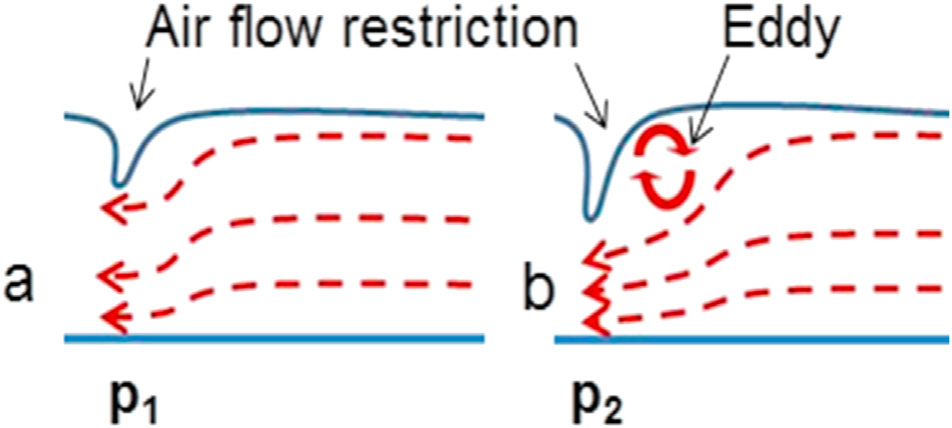
A simple schematic albeit exaggerated illustration of the effect of flow restriction on flow profile. A greater protrusion of the flow obstacle into the flow path in (b) generates an eddy (turbulent flow). In otherwise equal flow conditions between (a) and (b), the pressure, P2 (b) is greater than the pressure, P1 (a) as a result of small cross-sectional area at the restriction point. This difference is further magnified due to the presence of the turbulent flow.

Although several studies have shown anatomical narrowing of the pharyngeal airway due to expansion of the surrounding soft tissues, there are limited studies that describe the distribution of airflow and structural resistance that occurs in OSA. Through finite element modeling (FEM), computational fluid dynamics (CFD) can be used to simulate airflow dynamics and pressure distributions in a 3D format to understand the biomechanical properties of airway involved in OSA and to evaluate the various treatment outcomes on airway restriction of OSA (Mylavarapu et al. 2009, 2013; Zhao et al., 2013). Therefore, the present study was designed to illustrate the airflow dynamics within nasal and oral pharyngeal airways in obese/OSA and non-obese/non-OSA Yucatan minipigs with and without OSA. Yucatan minimigs were chosen for this study because they have been reported to present with OSA and display similarities in size, architecture, and tissue type between their pharyngeal airways and those of humans (Deng et al., 2020; Lonergan et al., 1998; Yang et al., 2004). There are also similarities in the tongue shape and size between Yucatan minipigs and humans (Herring et al., 2011). A decrease in the volume of pharyngeal airway passages is believed to increase the airway resistance and pressure that would cause a disruption the inspiratory laminar airflow as it enters the airway, causing the airflow to become turbulent (Ebben et al., 2016; Jeong et al., 2007). This turbulence created by an increased pressure is presumed to prevent the air from continuing down the airway (Figure 1). Therefore, the present study aims to bring insight into the mechanism that is responsible for the temporary cessation of airflow found in OSA. The hypothesis is that the restricted pharyngeal airway configuration in OSA/obese minipigs leads to higher resistance and pressure, which in turn produces local turbulence that leads to the temporary blockage of pharyngeal airway patency.

## 2 Materials and methods

### 2.1 Identification of sleep apnea/hypopnea and assessment of respiratory function

Five 8-11-month old Yucatan minipigs (S & S Farms, CA) were used for this study and underwent real-time natural sleep monitoring. Of these 5 minipigs, 3 were non-obese controls with body mass index (BMI) less than 39 (37.91–38.39), and 2 obese ones with BMI greater than 50 (50.13 and 51.42). The sleep monitoring sessions included the real-time recordings of either natural or sedated sleep for 3–4 h via two sets of wireless BioRadio systems (Great Lakes NeuroTechnologies, Valley View, OH). The detailed procedures for monitoring of natural and sedated sleep and the characterization of sleep apnea/hypopnea episodes were reported elsewhere (Deng et al., 2020).

**Figure 2:**
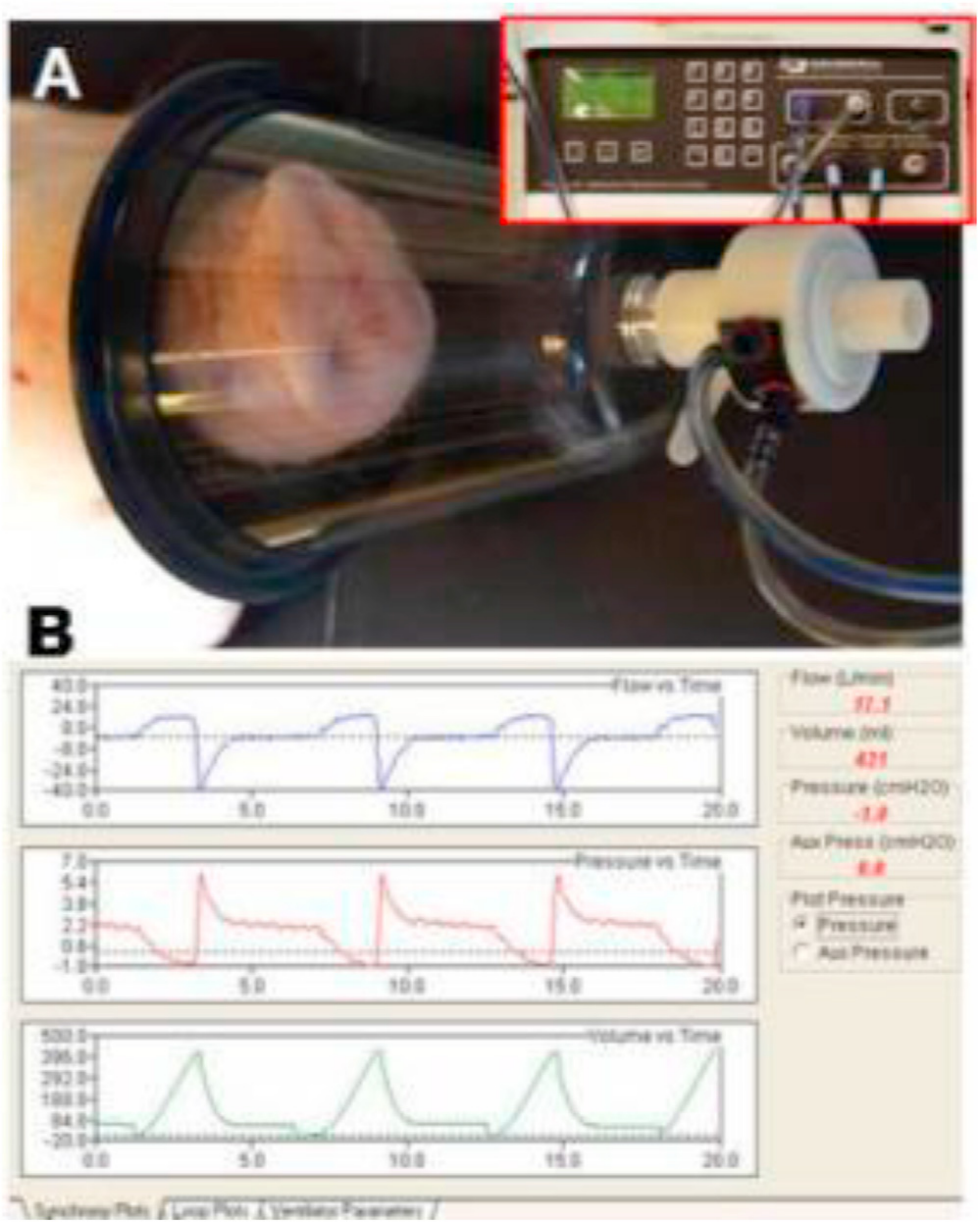
A: Placement of the facemask on the snout of the minipig under sedated sleep and the Research Pneumotach System sensor used to monitor and record airflow parameters. B: Raw Pneumotach tracings showing the recorded airflow parameters. Top, middle and lower panels indicate respisratory air flow, pressure, and volume *vs.* time, respectively.

Two to three days after sleep monitoring, all five minipigs were sedated by using I.M injection of xylazine (4 mg/kg), midazolam (0.5 mg/kg) and butorphanol (0.3 mg/kg). The respiratory functions were monitored and recorded for 15–20 min using the Research Pneumotach system (Hans Rudolph Inc. Shawnee, KS) through a facemask (Figure 2). These recorded airflow parameters were compared between each obese/OSA and non-obese/non-OSA animal and selected for the simulation of the CFD model described below.

All procedures of animal experiment were approved by the IACUC of University of Washington (Protocol # 3393-04).

### 2.2 MRI imaging and measurements of nasal and oral pharyngeal airway spaces

Under the same sedation procedures, MRI images (3T Philips Achieva Quasar Dual 3.0T whole body scanner with the Philips Extended MR Workspace Workstation) were obtained when the pigs were placed in prone position on the MRI scan table, as this is the common sleeping position in pigs (Deng et al., 2020). The MRI scan failed in one non-obese minipig #917, thus only four minipigs were included in the MRI results.

**Figure 3:**
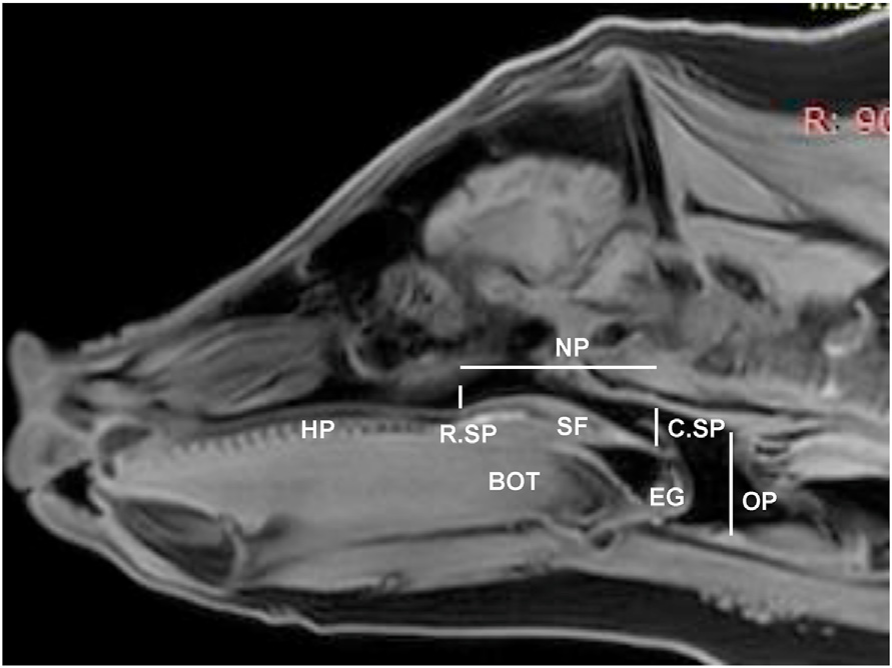
VISTA MRI sagittal slice by RadiAnt DICOM Viewer to measure dimensions of nasal and oral pharyngeal airway spaces. HP: Hard palate; SF: Soft palate; BOT: Base of the tongue; EG: Epiglottis. Two vertical lines indicate the rostral and caudal ends of soft palate (R.SP and C.SP). Horizontal and vertical dotted lines indicate the locations of nasal and oral pharyngeal airway spaces (NP and OP), respectively.

The images from the MRI Vista sequence with the resolution of 0.275mm were used to create the 3D configurations of the nasal and oral pharyngeal airway spaces. Each set of 2D MRI slices for each pig were retrieved and reviewed using RadiAnt DICOM Viewer (Medixant, Poznan, Poland) to identify the structures within the airway spaces. Then, RadiAnt DICOM Viewer was used to measure the distance between the ventral and dorsal borders of the airway spaces between the rostral and caudal ends of the soft palate, and the beginning of the trachea caudal to the epiglottis. These measurements provided airway dimensions of the nasopharynx at the beginning and end of the soft palate, and the oropharynx caudal to the soft palate and epiglottis (Figure 3).

**Figure 4:**
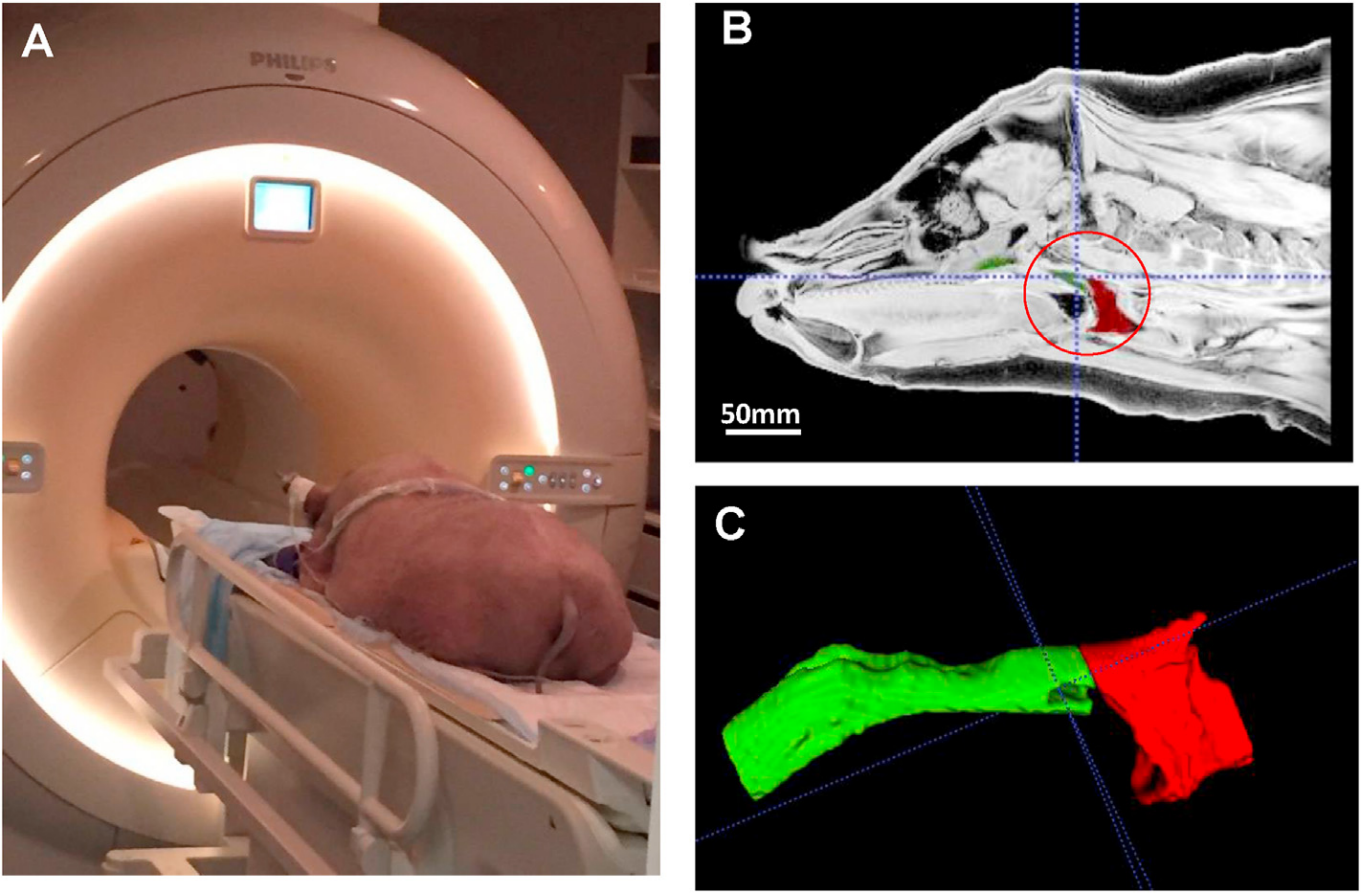
A: MRI imaging in the prone position under sedation. B: Sagittal slice of MRI image. The red circle indicates the region of interest (ROI). C: The segmented 3D model of the nasopharyngeal (green) and the oropharyngeal (red) airway.

The ITK-Snap software merged the 2D MRI slices together to create 3D configurations of nasal and oral pharyngeal airway spaces in the X, Y, and Z coordinates. The region of interest (ROI) was defined as the same as the airway dimension measurements: i.e., the rostral edge of the soft palate served as the anterior border, the beginning of the trachea served as the posterior border, the soft palate to the trachea as the ventral border, and the pharyngeal wall as the dorsal border. Within these boundaries, ITK-Snap was used to divide the volume of the ROI into 3D cubic voxel units. The volume within each voxel was integrated and then amalgamated together to form a 3D configuration of the airway spaces within the ROI. Then, ITK-Snap was used to segment the 3D configurations of the airways spaces by dividing them into the nasal (anterior) and oral (posterior) pharyngeal portions to isolate the location of possible turbulence in CFD modeling. The nasal portion spans from the rostral to the caudal portion of the soft palate, whereas the oral portion spans from the end of the soft palate to the start of the trachea (Figure 4).

### 2.3 CFD model simulations and analyses

The 3D configurations created in ITK-Snap were used for the CFD simulation. The CFD simulation consisted of 3 major steps: 1) converting 3D images into mesh frames; 2) applying governing equations and boundary conditions to the 3D mesh frames to simulate airflow pattern and velocity; and 3) post-simulation processing. All of the meshing conversions and CFD simulations were carried out using the Ansys 18.0 software package (Ansys Inc. PA). Details of these steps are described as follows: once the 3D airway space configurations were created, they were converted to *stl* files and imported into Ansys SpaceClaim 18.0 software, which converted the *stl* files into CAD files. The CAD files were subsequently loaded onto Ansys Fluent 18.0 and converted to mesh files using the CAD faceting algorithm. The algorithm detects surface facets and defines them as surface domain, which was used to generate a surface mesh. For the CFD analysis, the 3D configurations of pig #981 and #954, representing non-obese/non-OSA and obese/OSA minipigs respectively, were converted into meshes. These two configurations were chosen based on the drastic differences in soft palate and pharynx volumes, and therefore, selected as candidates for simulations. For inhalation and exhalation phases of respiration during sedated sleep, the governing equations for the laminar flow conditions were solved numerically using the Navier Stokes equations and the Reynolds Averaged Navier-Stokes (RANS) equations to test turbulent flow. The inhale and exhale air speeds measured by Research Pneumotach System were applied to the boundary conditions and were designated as V_0_ (Charts 1 and 2): no slip condition along the walls of the air pathway was assumed, i.e., air velocity immediately adjacent to the walls was 0 L per minute (LPM).

### 2.4 Data processing and analysis

From the recordings obtained from the Pneumotach, 30–50 consecutive respiratory cycles from each animal were selected and their tidal volumes and airflow velocities during inspiratory and expiratory phases were compared between each non-obese/non-OSA and obese/OSA animal. The measurements for the nasal and oral pharyngeal airway dimensions and volumes were not adjusted by the body size because human study has demonstrated that obesity does not lead to a larger airway. Instead, there seems to be a correlation between a decrease in airway with an increase in BMI (D'Anza et al., 2015). Due to the limited number of minipigs, no statistical tests were performed for the measurements of airway sizes.

## 3 Results

### 3.1 OSA/hypopnea episodes during sedated and/or natural sleep

Physical parameters of 5 minipigs are listed in Table 1. The criteria for the verification of OSA was reported elsewhere (Deng et al., 2020). In summary, of the 3 non-obese minipigs, only one (#716) showed a few >5s hypopnea episodes (apnea/hypopnea index (AHI) = 5) during sedated sleep. Both obese minipigs showed multiple >5s apnea/hypopnea episodes (AHI = 32–35) during both natural and sedated sleep, along with heavy snoring (Deng et al., 2020).

**Table 1:** Physical parameters of minipigs.

Pig No.	Age	Sex	BW(Kg)	BL (cm)	BMI(Kg/m^2^)	NC(cm)
**Obese Yucatan**						
930	8.5 M	M	68	115	51.42	82
954	8.5 M	F	71	119	50.13	77
**Non-obese Yucatan**						
716	8.0 M	F	45.9	110	37.93	61
970	9.5 M	M	55.0	120	38.19	66
981	11.0 M	F	55.1	119	38.91	74

BW: Body weight.

BL: Body length; measured from the tip of snout to the base of tail.

BMI: Body mass index, calculated at body weight (Kg)/body length(m)2.

NC: Neck circumference, measured at the location of thyroid cartilage.

### 3.2 Airway space sizes and respiratory parameters

**Figure 5:**
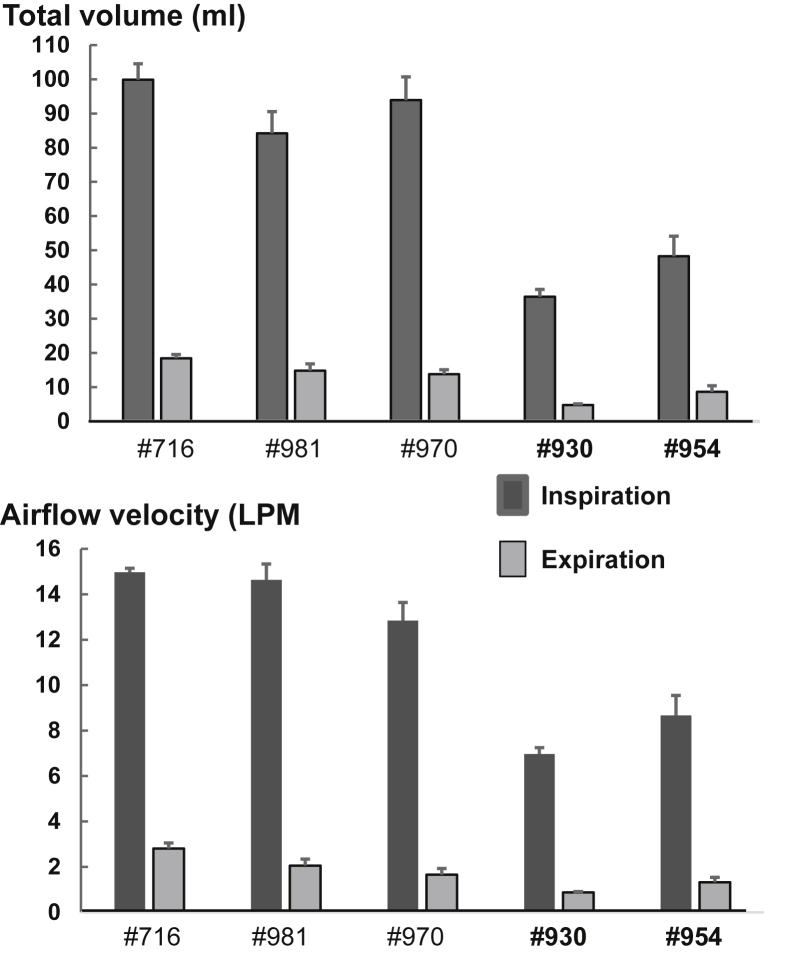
Comparisons of respiratory airflow parameters. A: Tidal volume of inspiration and expiration (Milliliter, ml); B: Airflow speed of inspiration and expiration (Liter Per Minute, LPM). Bolded pigs' numbers: obese OSA minipigs.

The 2D and 3D volumetric measurements of nasal and oral pharyngeal airway spaces are summarized in Table 2. Although much heavier body weights are shown in Table 1, airway dimensions of obese/OSA minipigs were either similar or smaller to those of non-obese/non-OSA controls, particularly obese #954, and the obese/OSA minipigs also showed smaller volumes in both nasal and oral pharyngeal airway spaces. Furthermore, the obese/OSA minipigs presented with much lower tidal volumes of both inhalation (92.66 *vs* 41.34 mL) and exhalation (15.67 *vs* 7.81 mL), and lower inspiratory airflow speed (14.15 *vs* 7.81 LPM) compared to the non-obese/non-OSA controls (Figure 5).

**Table 2:** Dimensional and volumetric measurements.

Pig #	Dimension (mm)			Volume (mm^3^)		
	R.SP	C.SP	OP	NP	OP	Total
716	10.17	8.46	29.84	10.10	8.68	18.78
981	8.89	14.18	37.05	13.91	16.64	30.55
**930**	**10.05**	**8.94**	**29.27**	**9.53**	**7.84**	**17.37**
**954**	**8.49**	**6.67**	**22.09**	**8.17**	**8.45**	**16.62**

OP: Oral pharyngeal airway; NP: Nasal pharyngeal airway; Bolded: Obese minipigs.

Bolded: Obese minipigs.

### 3.3 CFD simulations

**Figure 6:**
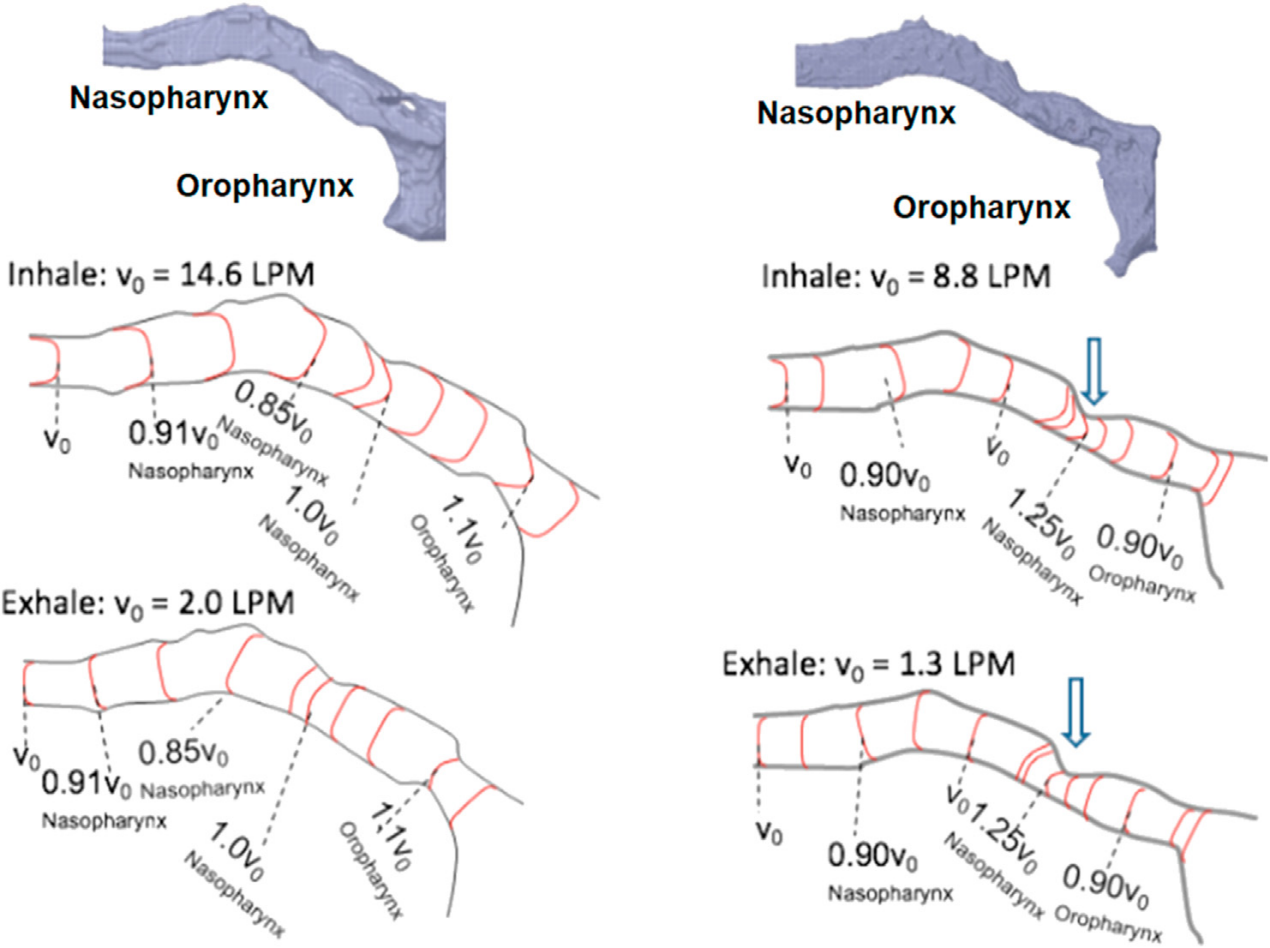
Comparisons of airflow velocity profiles along the nasal and oral pharyngeal airways between the representatives of the non-obese/non-OSA (#981, left column) and obese/OSA (#954, right column) minipigs. A: Nasal and oral pharyngeal airways; B: Airflow profiles during the inspiratory phase; C: Airflow profiles during the expiratory phase. Please note that although turbulence is absent in airways of both CFD models, the airway of OSA/obese minipig displayed an increased airflow speed at the narrowest portion of the nasopharynx (vertical arrows). LPM: Litter Per Minute.

Using the applied boundary conditions, the airflow velocity profiles along the dimensions of nasal and oral pharyngeal airways were calculated for the phases of inhalation and exhalation in both non-obese/non-OSA and obese/OSA minipig models. No turbulence was observed in either model. The velocities generally varied with respect to the width of the airflow pathway where a narrower pathway correlated to a higher velocity. The velocity zero (V_0_) was designed as no slip conditions along the walls of the airflow pathway assumed, i.e., when the airflow first reached the wall of the pharyngeal airway. The velocity variations ranged from -15% to +10% of V_0_'s in both inhalation and exhalation for the non-obese/non-OSA minipig. In the obese/OSA minipig, the range was -10% to +25% of V_0_'s, where the 25% increase was observed at the narrowest part of the airflow pathway located in the end of nasal pharynx (caudal end of soft palate), as indicated by the vertical arrows (Figure 6).

## 4 Discussion

The pharyngeal airway is a complicated structure and plays an important role in several critical functions such as swallowing, speech, and breathing. From the respiratory perspective, the primary goal of the pharyngeal function is to keep the airway patent to allow airflow in and out of the lung. Airway patency is controlled by several muscle groups that vary in activity with sleep state, within each breath, and phase of respiration (Edwards and White 2011). Therefore, exploring the interaction between airway anatomy and its airflow dynamics is a critical to understand the respiratory physiology and pathophysiology.

OSA is characterized by repeated complete (apnea) or partial (hypopnea) reduction in pharyngeal airway patency (Pelteret and Reddy 2014). Therefore, both airway anatomy and its neuromuscular control have been implicated in the pathophysiology of OSA. Studies have shown that the structural change of narrowing the upper airway could additionally predispose the airway to collapse (Mylavarapu et al., 2013). Although the partial or complete collapse of pharyngeal airway is widely accepted as a primary cause of OSA, the etiology of the airway collapse is poorly understood and airflow dynamics leading to airflow reduction or blockage is hardly predicted. Therefore, image based CFD is an ideal modeling tool that can generate a wealth of data about the airway flow field and provide important insight regarding the pathophysiology of OSA. CFD has been used to characterize the airflow in various airway models reconstructed from MRI or CT images, which mostly used laminar or steady Reynolds-Averaged Naivier-stroke (RANS) model as used in the present study. Given the great variations in the shape and size of airway and changes in relation to various physiological or pathological conditions, these numerical algorithms need to be simulated and validated by actual respiratory airflow parameters in order to accurately predict the airflow dynamics within the airway from animal experiments or clinic setting (Allen et al., 2004; Mylavarapu et al., 2009; Ogisawa et al., 2019; Zhao et al., 2013). This emergent approach of image-based CFD has been applied for preoperative prediction of airway changes after maxillomandibular advancement surgery (Ogisawa et al., 2019) and assessment of upper airway response to oral appliance treatment for OSA (Zhao et al., 2013). However, in the majority of these image-based CFD studies, the simulation of the model has been established by the respiratory airflow parameters measured from an identical or similar physical model rather than the modeled subject. In the present study, the CFD models were established using meshing 3D segmented data from MRI images. These models were simulated and validated using the inspiratory and expiratory airflow velocity from the same representative of obese/OSA and non-obese/non-OSA minipigs as those for the model establishment. Thus, the present study provides the validated CFD models to depict airflow dynamics in the nasal and oral pharyngeal airways in normal and obese/OSA minipigs.

The present study shows that obese/OSA minipigs presented smaller tidal volume and slower airflow velocity of both inspiration and expiration phases compared to non-obese/non-OSA controls (Figure 5). The FEM simulation identified a ~25% increase of airflow velocity at the center of the narrowest portion of pharyngeal airway located at the transitional region of nasal and oral pharynx. However, the overall volumetric flow rate is decreased by 30% (Figure 6). This contradiction should attribute to the methods of the actual airflow velocity measurement using Peumotach system and FEM simulation. In the former, the velocity was measured by the sensor located at the end of mask (Figure 2), but in the latter, the velocity was measured at each meshed location of pharyngeal airway (Figure 6).

The CFD model of the present study identified that the narrowest portion of pharyngeal airway in obese/OSA minipig is located at the transitional region of the nasal and oral pharynx, i.e. the caudal end of soft palate (retropalatal) and beginning of oral pharynx (retroglossal). This finding is consistent with a number of clinical studies (Simpson et al., 2018). Therefore, various surgical treatments have been focused on reducing the tissue mass of this region for airway expansion (Mulholland et al., 2019) and the study also found that this region was mostly affected by the type of masks (Ebben et al., 2016).

Turbulence would have effectively contributed to an added resistance to the overall airflow, thereby reducing the air volume exchange during respiration in obese/OSA minipigs. However, the observed airflow patterns were laminar in nature for both the non-obese/non-OSA control and obese/OSA minipigs. This observation may lead to the hypothesis that turbulence is expected in the obese/OSA minipigs due to abnormal variations in the pharyngeal airway. The reasons for the lack of turbulence observed are two-fold. The formation of turbulence requires a combination of two parameters: an abrupt dimensional change in the airflow pathway and sufficiently high airflow velocity. In the case of the obese/OSA minipig, a narrower gap was seen in the pharyngeal airway, resulting in a larger velocity gradient across the gap 25% (vertical arrow in Figure 6). However, this narrowing and the subsequent expansion along the airflow path are not abrupt enough for the given inhale and exhale airflow velocities to create turbulence. Despite the increased velocity in this local region, the airflow tidal volume and overall airflow velocity were much lower in obese/OSA minipigs compared to the non-obese minipigs (Figure 5).

With the lack of turbulence observed in obese/OSA minipigs, the lower air volume exchange during respiration and airway collapse during apnea/hypopnea episodes is likely the result of a lack of “air pumping efficiency” to compensate for the narrower air pathway. Since a smaller cross section restricts the volumetric airflow, the narrower airflow pathway reduces the total volumetric airflow. Although there is some compensation in the increased airflow velocity locally through the narrowest region of the nasopharynx, the volumetric airflow rate remains reduced. In obese/OSA minipigs with a narrower airflow pathway, the volumetric airflow rate would naturally be lower than non-obese/non-OSA controls unless their lungs pumped more air to compensate for the reduced airflow rate. The fact that the measured tidal volumes and inspiratory airflow velocity were significantly lower in obese/OSA minipigs indicated that its lungs were not pumping fast enough for its airflow volume to match that of the controls.

Hence, the combination of measured airflow parameters and airflow characteristics simulated by CFD provided the following insights. The narrowing of the airflow pathway in obese/OSA minipig does not contribute to any turbulent airflow, which would have resulted in added airflow resistance during respiration and contributed to the airway collapse. Rather, the drop in the air volume exchange in obese/OSA minipigs relative to the non-obese controls is due to the added resistance by the narrower air pathway. In conjunction with the narrower air pathway in obese/OSA minipigs, their lungs are not strong enough to overcome this resistance to match the airflow rate of non-obese controls.

Although the overall airflow rate of obese/OSA minipigs resulted in a significant decrease in the air volume exchange relative to the controls, the CFD simulation identified an increased airflow speed in the nasopharyngeal airway. This increase in airflow speed was isolated in the narrowest portion of the nasopharynx. By identifying the presence of an increase in airflow speed and isolating its location, this CFD simulation provides the framework for succeeding OSA research. In particular, this analysis of increased airflow speed will be influential in research that aims to prevent the airway collapse in OSA.

There are several limitations in this study. First, the sample size was small so some endpoints with lesser significance may not have been identified, and this may also weaken the clinical significance of this study. Second, even though no animal model perfectly simulates humans, there are several differences in the spatial relationships in airway structures between pigs and humans. Unlike humans, the larynx does not descend, and the tongue base is not a part of airway in pigs. However, in the pigs the tongue base strongly influences the airway through its contact with the soft palate and the pig sleeps in both the prone position and on its side (Lonergan et al., 1998). Therefore, the results of the current study may not be directly applied in human OSA *per se*. Nevertheless, the results explain how the airway collapse in obese OSA minipigs could occur, which may refer to potential reason of airway collapse in human OSA as well. Third, we did not examine the activity of pharyngeal muscle and did not obtain the airway images during natural sleep, although the sedated sleep for MRI scan in the resent study is similar to the natural sleep (Deng et al., 2020) Finally, the CFD model assumes that the airway tissues are stationary during each phase of respiration and does not model the dynamic airway collapse.

## 5 Conclusions

Obese/OSA Yucatan minipigs present the reduced dimension of the nasal pharyngeal airway and respiratory tidal volumes, and increased airflow velocity. However, the results of the FEM simulation indicated that turbulence was not produced in the pharyngeal airway despite the local higher airflow velocity was identified in the narrowest part of the airflow pathway located in the end of nasal pharynx (caudal end of soft palate).

## Declarations

### Author contribution statement

Zi-Jun Liu: Conceived and designed the experiments; Performed the experiments; Wrote the paper.

Tiffany Do: Analyzed and interpreted the data; Wrote the paper.

Hanson Fong: Contributed reagents, materials, analysis tools or data.

### Funding statement

Zi-Jun Liu was supported by 10.13039/100000072National Institute of Oral and Craniofacial Research, Bethesda, MD, United States, Grant R21 DE023988. Zi-Jun Liu and Tiffany Do were supported by Morell Fund for the Summer Research Fellowship (SURF), School of Dentistry, University of Washington, Seattle, WA, United States.

### Data availability statement

Data included in article/supplementary material/referenced in article.

### Declaration of interests statement

The authors declare no conflict of interest.

### Additional information

No additional information is available for this paper.
